# Impact of Chronic Pain on Caregiving Tasks for Dementia Family Caregivers

**DOI:** 10.1093/geroni/igaf122.2979

**Published:** 2025-12-31

**Authors:** Aryn Lee, Shelbie Turner, Karl Pillemer, M Carrington Reid

**Affiliations:** Cornell University Medical College, New York, New York, United States; Weill Cornell Medicine, New York, New York, United States; Cornell University, Ithaca, New York, United States; Weill Cornell Medicine, New York, New York, United States

## Abstract

Recent evidence suggests pain is highly prevalent among dementia family caregivers, but questions about how caregivers’ pain impacts care quality remain unanswered. Accordingly, we used a nationwide sample of dementia family caregivers with chronic pain to identify the extent to which caregivers attribute caregiving difficulty to pain. Caregivers (n = 269) reported whether they were responsible for helping care recipients with ten basic and instrumental activities of daily living (BADL & IADL), and whether they experienced difficulty completing each task. If they did report difficulty, they then rated how much of that difficulty they attribute to their pain. We first ran descriptive statistics to determine the prevalence of care task difficulty and mean pain-attributed difficulty and then ran ANOVA models to assess for between-group differences (e.g., racial differences) in pain-attributed difficulty with the care tasks. 205 caregivers helped their care recipient with at least one BADL and reported difficulty with an average of 2.65 BADLs, while 263 helped with at least one IADL and reported difficulty with an average of 2.90 IADLs. When asked about the extent to which pain contributed to the difficulty helping care recipients with BADL/IADLs, caregivers’ average response was 6.81 for BADLs and 6.49 for IADLs. Compared to White caregivers, Black caregivers attributed less difficulty with both BADLs and IADLs to pain. Pain appears to be a major contributor to the difficulty dementia family caregivers experience when helping care recipients with daily activities, suggesting that pain is not only highly prevalent but also highly consequential to caregiving outcomes.

